# Free Radicals and Antioxidant Status in Protein Energy Malnutrition

**DOI:** 10.1155/2014/254396

**Published:** 2014-03-27

**Authors:** M. Khare, C. Mohanty, B. K. Das, A. Jyoti, B. Mukhopadhyay, S. P. Mishra

**Affiliations:** ^1^Department of Anatomy, Institute of Medical Sciences, Banaras Hindu University, Varanasi, Uttar Pradesh, India; ^2^Department of Pediatrics, Institute of Medical Sciences, Banaras Hindu University, Varanasi, Uttar Pradesh, India; ^3^Department of Biochemistry, Institute of Medical Sciences, Banaras Hindu University, Varanasi, Uttar Pradesh, India

## Abstract

*Background/Objectives*. The aim of this study was to evaluate oxidant and antioxidant status in children with different grades of Protein Energy Malnutrition (PEM). *Subjects/Methods*. A total of two hundred fifty (250) children (age range: 6 months to 5 years) living in eastern UP, India, were recruited. One hundred and ninety-three (193) of these children had different grades of PEM (sixty-five (65) children belong to mild, sixty (60) to moderate, and sixty-eight (68) to severe group). Grading in group was done after standardization in weight and height measurements. Fifty-seven (57) children who are age and and sex matched, healthy, and well-nourished were recruited from the local community and used as controls after checking their protein status (clinical nutritional status) with height and weight standardization. Redox homeostasis was assessed using spectrophotometric/colorimetric methods. *Results*. In our study, erythrocyte glutathione (GSH), plasma Cu, Zn-superoxide dismutase (Cu,Zn-SOD,EC 1.15.1.1), ceruloplasmin (Cp), and ascorbic acid were significantly (*P* < 0.001) more decreased in children with malnutrition than controls. Plasma malondialdehyde (MDA), and protein carbonyl (PC) were significantly (*P* < 0.001) raised in cases as compared to controls. *Conclusion*. Stress is created as a result of PEM which is responsible for the overproduction of reactive oxygen species (ROSs). These ROSs will lead to membrane oxidation and thus an increase in lipid peroxidation byproducts such as MDA and protein oxidation byproducts such as PC mainly. Decrease in level of antioxidants suggests an increased defense against oxidant damage. Changes in oxidant and antioxidant levels may be responsible for grading in PEM.

## 1. Introduction

Malnutrition is one of the major public health challenges in developing countries. Usually is referred to as a silent emergency as it has devastating effects on children, society, and future mankind. The net loss of body protein particularly skeletal muscle protein is likely to be a major factor responsible for PEM [1, 2]. Plasma albumin [3, 4], erythrocyte glutathione, and other endogenous antioxidant molecules such as bilirubin and uric acid [5] directly scavenge ROSs. Dietary deficiency of protein not only impairs the synthesis of plasma albumin and antioxidant enzymes but also reduces tissue concentrations of antioxidants, thereby resulting in a compromised antioxidant status [6, 7]. Copper-zinc and manganese are indispensable metals for the activities of Cu-Zn-SOD and Mn-SOD, respectively. Free radicals are very short lived and unstable, so they are difficult to measure. But their detrimental effects can be measured by estimating their byproducts. Markers of oxidative stress are MDA, a by-product of lipid peroxidation and PC, a byproduct of protein oxidation. Defense capacity against ROS can be measured blood levels of GSH, glutathione peroxidase (GPx), Cu,Zn-SOD, Cp, and ascorbic acid. The pathogenesis of extreme muscle wasting (emaciation) and anemia commonly found in children with PEM has been suggested to be caused by an imbalance between the production of these toxic free radicals and antioxidant potential [8]. Very few studies of oxidant and antioxidant status in PEM children have been done so far. Therefore the aim of present study is to explore the status of oxidants and antioxidants in grades of PEM.

## 2. Subjects and Methods

The study was conducted in the Department of Biochemistry and the Department of Pediatrics, SSLH, Institute of medical sciences, Banaras Hindu University, Varanasi. 250 children aged between 6 months to 5 years were selected. These children were examined for malnutrition, diagnosed, and classified according to nutrition subcommittee of IAP in 4 grades with various percentages of expected body weight for age [9].

All the chemicals and reagents required for the analysis were of analytical grade, and proper aseptic measures had been taken while study. Estimation was done by Spectrophotometer. The children were classified using the standard value, that is, 100% as 50th percentile of the standard NCHS growth standard, Normal > 80% of standard weight for age. Grade-I = 71–80%, Grade-II = 61–70%, Grade-III = 51–60%, and Grade IV = < 50%. According to this classification, 193 children were of strictly defined malnutrition cases; of these children, 65 belong to grade-I, 60 to grade-II, and 68 to grade-III, and none of the cases was of grade IV. 57 normal and healthy children presenting no clinical and anthropometric signs or symptoms suggestive of any form of malnutrition with age and sex matched were used as control group. The gradation was done on the basis of clinical examination and plasma protein level was not assayed. Male and female ratio was 5 : 4 in both case and control groups. The hemoglobin level of the control group was about 11.9 gm/dL (conventional unit, estimated by Drabkin's method) and hemoglobin levels in grade 1, grade 2, and grades (3 + 4) were about 11.5 gm/dL, 10.2 gm/dL, 8.41 gm/dL, respectively. Ethical clearance to conduct the present study was obtained from the ethical committee Institute of medical sciences, BHU. Informed consent was taken from the attendants of the patients. Blood samples were collected from strictly defined malnutrition cases and from normal subjects under aseptic condition. Random blood samples were taken from the patients attending the paediatric OPD of the Hospital (between 8AM and 2PM). Children suffering from severe infections, edema, taking micronutrient, and antioxidants supplement were excluded from the study. All patients and controls were asked about the history concerning their diet, and clinical examination was done for their anthropometric measurements. Five mL of venous blood was sampled from each subject. Three mL of blood was allowed for 30–60 minutes for spontaneous blood clotting. The serum was separated from the blood cells by centrifugation at 3000 rpm for 10 minutes at room temperature. The serum was decanted and centrifuged twice for 5 minutes at 3000 rpm to remove any blood cell remnants, decanted again, and then stored at −20°C in deionized eppendorf tube vials until assay. Two mL of whole blood in EDTA was stored separately for glutathione estimation and was stored at −20°C without any preservative. The red blood cells were lysed before estimating glutathione estimation. Oxidants such as MDA and PC were assayed by the thiobarbituric acid test [10] and Reznik and Packer [11], while antioxidants such as ascorbic acid, Cu,Zn-SOD, Cp, and glutathione levels by Roe [12]; S. Marklund and G. Marklund [13]; Ravin [14] and Beutler et al. [15], respectively. Statistical analysis was performed by one way analysis of variance (ANOVA), Post hoc analysis (Bonferroni test) and Pearson correlation coefficients using SPSS 11.5 software. Subjects with malnutrition were compared with nonmalnourished controls. The level of significance was considered at *P* < 0.05.

## 3. Results

**Figure 1:**
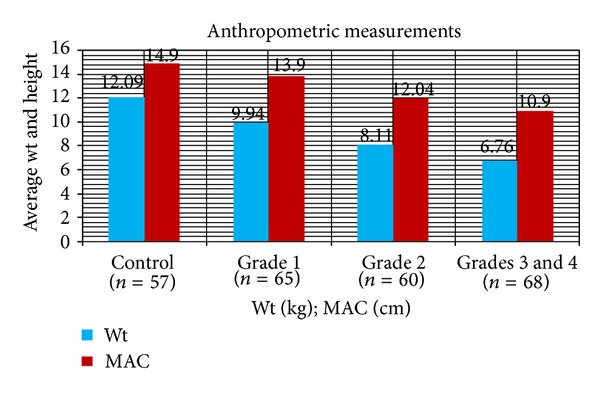
Anthropometric measurements in cases of PEM and Control. Results are expressed as mean ± S.D. *P* < 0.01 for Wt. and *P* < 0.001 for MAC while comparing Wt. and MAC of PEM (cases) with control by ANOVA test.

**Figure 2:**
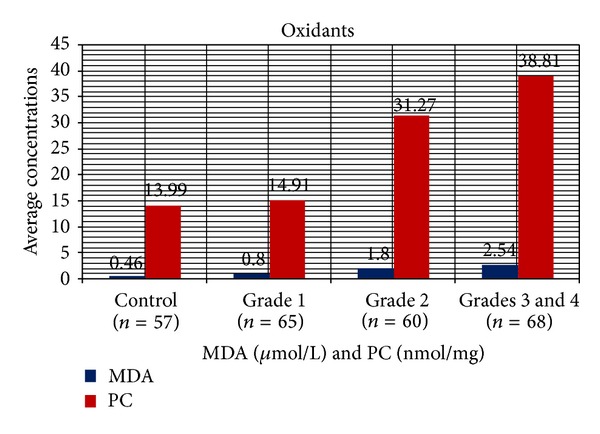
Serum MDA and PC conc. in PEM (cases of different grades, i.e., 1, 2, and 3) and control measured by thiobarbituric acid test [10] and Reznick and Packer [11] method, respectively. Results are expressed as mean ± S.D. *P* < 0.001 by ANOVA test.

**Figure 3:**
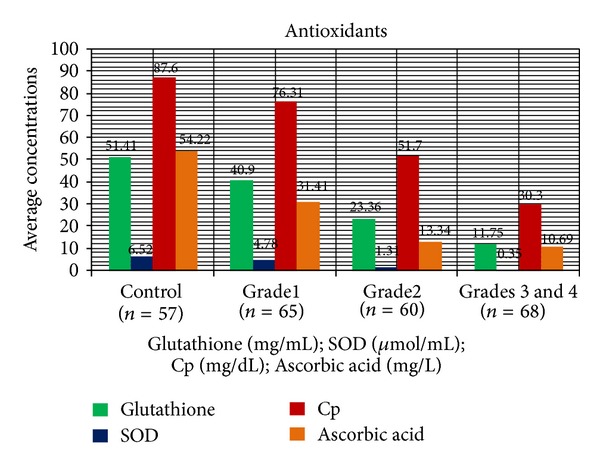
Glutathione; CU,ZN-SOD; Cp; ascorbic acid conc. in PEM (cases of different grades i.e, 1, 2, and 3) and control measured by Beutler et al. 1963 [15]; S. Marklund and G. Marklund [13]; Ravin [14]; Roe [12]; method, respectively. Results are expressed as mean ± S.D. *P* < 0.001 while comparing PEM (cases) with control by ANOVA test.

**Figure 4:**
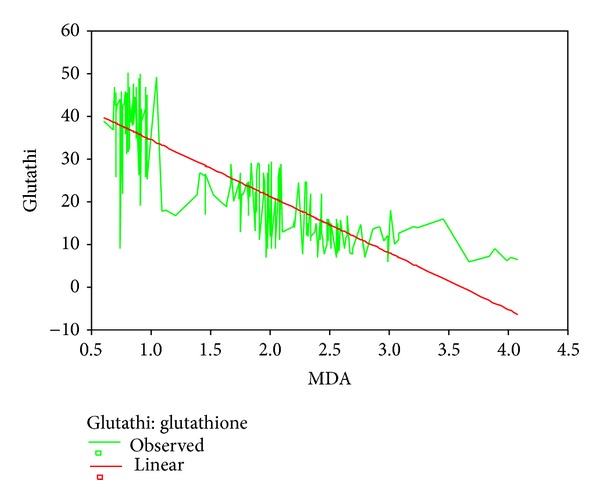
Correlation between GPX and Serum MDA in protein energy malnutrition (*P* < 0.001).

**Figure 5:**
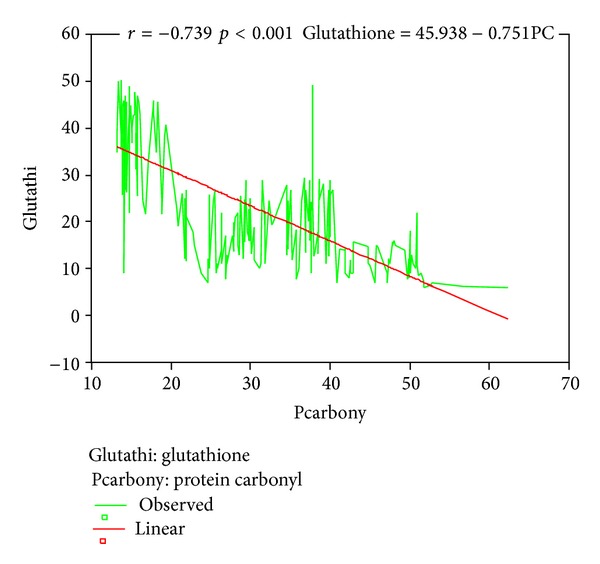
Correlation between GPX and Serum Protein Carbonyl in protein energy malnutrition (*P* < 0.001).

**Figure 6:**
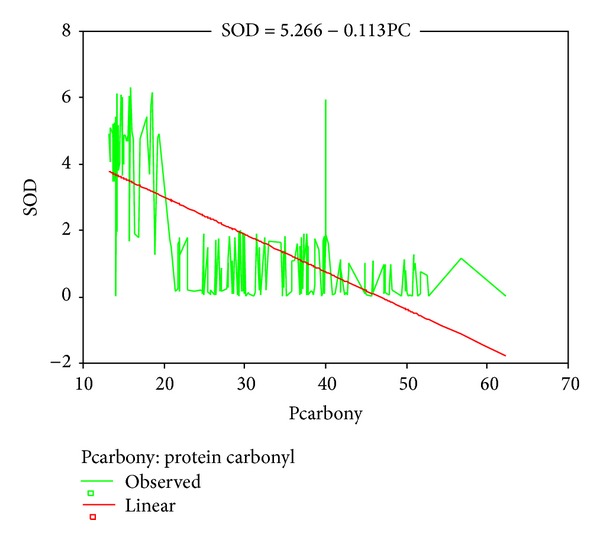
Correlation between SOD and Serum Protein Carbonyl in protein energy malnutrition (*P* < 0.001).

**Figure 7:**
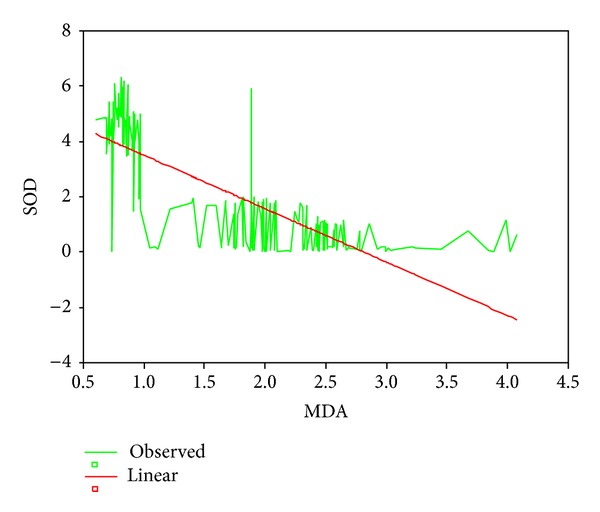
Correlation between SOD and Serum MDA in protein energy malnutrition (*P* < 0.001).

Mean age, head circumference (HC), and chest circumference (CC) between malnourished and control groups were compared. Weight, height, and Mid arm circumference (MAC) were significantly reduced in malnourished children (Table 1; Figure 1). The mean oxidant damage products (MDA and PC) levels were significantly increased in malnourished group (*P* < 0.001) (Table 2; Figure 2) while the antioxidants (Cu,Zn-SOD, Cp, GSH, and ascorbic acid) were significantly reduced (Table 3; Figure 3). Significant negative correlations were observed between MDA and antioxidants (Cu,Zn-SOD, glutathione, ceruloplasmin, and ascorbic acid) and PC and antioxidants (Table 4). Correlation between GPX and serum MDA in protein energy malnutrition (*P* < 0.001) in Figure 4, correlation between GPX and serum protein carbonyl in protein energy malnutrition (*P* < 0.001) in Figure 5, correlation between SOD and serum protein carbonyl in protein energy malnutrition (*P* < 0.001) in Figure 6, correlation between SOD and serum MDA in protein energy malnutrition (*P* < 0.001) in Figure 7.

## 4. Discussion

In the present work, we examined the status of both antioxidant and oxidant activities. Malnourished children were found to have more oxidant damage products and less antioxidant levels. Alternatively, the control group consisting of healthy children had comparatively less oxidant damage product and more antioxidant level. ROSs degrades polyunsaturated lipids, forming MDA. Raised levels of lipid peroxidation products in the serum are used as a marker for tissue damage, and MDA is regarded as one of the most stable products of lipid peroxidation. In present study, there is a significant increase in serum MDA in malnourished children as compared to control (*P* < 0.001) (Table 2; Figure 2). Increased plasma MDA levels have been demonstrated previously by other workers also. Boşnak et al. [16] in 2010 conducted a study on the oxidative stress in marasmus children and concluded that MDA was significantly higher in marasmus children. In our present study, there was a significant increase in serum PC in malnourished children as compared to control (*P* < 0.001) (Table 2; Figure 2). PC is a byproduct of protein oxidation, and no related studies has been done earlier on PC in PEM children.

The plasma Cu,Zn-SOD level was found to be significantly decreased in cases. This supports its role as an antioxidant in cases of malnutrition where its level decreases to counteract the oxidative stress. In our present study however Cu,Zn-SOD level is more significant in grades III and IV. These results are in agreement with findings by Golden and Ramdath, 1987 [17]. However, Ashour et al. 1999 [18] had reported an increase of the antioxidant enzymatic activities in 40% of the marasmic children, whereas Sive et al. 1993 [19] found no changes in Cu,Zn-SOD level in marasmic children. In our present study, mean whole blood GPx activity is significantly decreased in malnourished children compared with control (*P* < 0.001) (Table 3; Figure 3). These results are in agreement with those reported by Ashour et al. in 1999 [18], Golden and Ramdath in 1987 [17], and Sive et al. in 1993 [19]. In our present study, there is significantly depressed plasma ceruloplasmin level (*P* < 0.01) (Table 3; Figure 3) which is in agreement with the study done by Ashour et al. in 1999 [18] who also showed lower plasma concentration of ceruloplasmin in children with malnutrition. This reduction of the ceruloplasmin may be due to its excessive loss or destruction or its inability to synthesis ceruloplasmin. The concentration of ascorbic acid was markedly depressed in the malnourished group (*P* < 0.001) (Table 3; Figure 3). These results are in agreement with the results reported by Ashour et al. in 1999 [18]. Therefore it appears that these biochemical alterations are indicative of oxidative damage in malnutrition. Negative correlations between oxidant (PC and MDA) and antioxidants (Cu,Zn-SOD, GPx, Cp, and ascorbic acid) (Table 4) indicate that the magnitude of initial oxidative stress was too high beyond the compensatory capacity of antioxidants. Aldehydes formed endogenously during lipid peroxidation such as MDA which reacts on cellular proteins to form adducts (ALEs) that induce protein dysfunctions and alter cellular responses [20]. Increased MDA levels in children with different grades of PEM may cause accelerated PC formation in plasma proteins especially in albumin. Increased oxidative stress may result from some deleterious effects of deficient caloric and micronutrient intake. In view of the reduced antioxidant defense capacity and the presence of increased oxidant stress, strategies should be developed to strengthen the antioxidant system of children with protein energy malnutrition, so as to prevent further damage. Further studies are required to determine the cause-and-effect relationship and its prognostic value in patients with malnutrition.

The PC and MDA were measured as an estimate of free radical damage. So they do not give directly the values of the free radicals present in the serum. For more accurate estimation of free radicals, Electron Spin Resonance (ESR) should be used.

## Figures and Tables

**Table 1:** Anthropometric measurements in cases of malnutrition and control.

Parameters	Mean ± SD				Intergroup comparison one way ANOVA	Post Hoc test significant pairs
	Control *n* = 57	Grade 1 *n* = 65	Grade 2 *n* = 60	Grades 3 and 4 *n* = 68		
Wt (kg)	12.09 ± 3.09	9.94 ± 2.28	8.11 ± 1.77	6.76 ± 2.04	*F* = 75.392 *P* < 0.01	All significant
Age (month)	29.52 ± 15.95	30.88 ± 15.33	27.77 ± 13.95	26.27 ± 17.64	*F* = 1.221 *P* > 0.05	
Ht (cm)	87.80 ± 12.03	88.76 ± 10.95	83.97 ± 10.69	80.91 ± 13.52	*F* = 7.001 *P* < 0.001	Control Grades 3 and 4 Grade 1 and Grades 3 and 4
HC (cm)	47.4 ± 2.18	47.7 ± 1.78	46.9 ± 2.10	46.8 ± 2.48	*F* = 2.507 *P* > 0.05	No group significant
MAC (cm)	14.9 ± 0.89	13.9 ± 0.86	12.04 ± 1.14	10.9 ± 0.97	*F* = 263.723 *P* < 0.001	All significant
CC (cm)	48.52 ± 3.59	49.02 ± 3.16	48.1 ± 3.10	47.7 ± 4.35	*F* = 1.803 *P* > 0.05	No group significant

**Table 2:** Oxidants in different grades of PEM.

Parameters	Mean ± SD				Intergroup comparison of one way ANOVA	Post HOC Test significant pairs
	Control	Grade 1	Grade 2	Grades 3 and 4		
MDA (*μ*moL/L)	0.46 ± 0.05	0.80 ± 0.07	1.80 ± 0.07	2.54 ± 0.52	*F* = 605.395 *P* < 0.001	All significant
PC (nmoL/mg)	13.99 ± 1.53	14.91 ± 1.48	31.27 ± 7.72	38.81 ± 10.24	*F* = 241.998 *P* < 0.001	All significant

**Table 3:** Serum antioxidants in cases of malnutrition and control.

Parameters	Mean ± SD				Intergroup comparison one way ANOVA	Post Hoc test significant pairs
	Control *n* = 57	Grade 1 n = 65	Grade 2 n = 60	Grades 3 and 4 n = 68		
Glutathione (mg/mL)	51.41 ± 4.52	40.90 ± 5.51	23.36 ± 5.0 0.59	11.75 ± 3.23	*F* = 1173.572 *P* < 0.001	All significant
SOD (*μ*moL/mL)	6.52 ± 0.72	4.78 ± 0.68	1.31 ± 0.89	0.35 ± 0.41	*F* = 1351.690 *P* < 0.001	All significant
Ceruloplasmin (mg/dL)	87.60 ± 8.21	76.31 ± 5.70	51.70 ± 9.69	30.30 ± 11.56	*F* = 593.930 *P* < 0.001	All pairs
Ascorbic acid (mg/L)	54.22 ± 8.46	31.41 ± 6.70	13.34 ± 2.94	10.69 ± 1.91	*F* = 1001.035 *P* < 0.001	All pairs

**Table 4:** 

Correlation between antioxidants and oxidants	Coefficient of correlation and its statistical significance between normal and malnourished
MDA and ceruloplasmin	*r* = −0.904, *P* < 0.001
MDA and Glutathione	*r* = −0.901, *P* < 0.001
MDA and CU, ZN-SOD	*r* = −0.869, *P* < 0.01
MDA and ascorbic acid	*r* = −0.821, *P*<0.001
Protein carbonyl and glutathione	*r* = −0.808, *P* < 0.01
Protein carbonyl and CU, ZN-SOD	*r* = −0.789, *P* < 0.01
Protein carbonyl and ascorbic acid	*r* = −0.727, *P* < 0.01
Protein carbonyl and ceruloplasmin	*r* = −0.851, *P* < 0.01
